# Different Lipid Signature in Fibroblasts of Long-Chain Fatty Acid Oxidation Disorders

**DOI:** 10.3390/cells10051239

**Published:** 2021-05-18

**Authors:** Khaled I. Alatibi, Judith Hagenbuchner, Zeinab Wehbe, Daniela Karall, Michael J. Ausserlechner, Jerry Vockley, Ute Spiekerkoetter, Sarah C. Grünert, Sara Tucci

**Affiliations:** 1 Department of General Pediatrics, Adolescent Medicine and Neonatology, Faculty of Medicine, Medical Centre-University of Freiburg, 79106 Freiburg, Germany; Khaled.ibrahim.alatibi@uniklinik-freiburg.de (K.I.A.); Zeinab.wehbe@uniklinik-freiburg.de (Z.W.); ute.spiekerkoetter@uniklinik-freiburg.de (U.S.); sarah.gruenert@uniklinik-freiburg.de (S.C.G.); 2 Faculty of Biology, University of Freiburg, 79104 Freiburg, Germany; 3 Department of Pediatrics II, Medical University Innsbruck, 6020 Innsbruck, Austria; Judith.Hagenbuchner@i-med.ac.at; 4 Department of Pediatric Hematology and Oncology, Faculty of Medicine, Center of Pediatric and Adolescent Medicine-Medical Center-University of Freiburg, 79106 Freiburg, Germany; 5 Department of Pediatrics I, Medical University Innsbruck, 6020 Innsbruck, Austria; Daniela.Karall@i-med.ac.at (D.K.); michael.j.ausserlechner@i-med.ac.at (M.J.A.); 6 School of Medicine, University of Pittsburgh, Pittsburgh, PA 15260, USA; vockleyg@upmc.edu; 7 Center for Rare Disease Therapy, UPMC Children’s Hospital of Pittsburgh, Pittsburgh, PA 15224, USA; 8 Graduate School of Public Health, University of Pittsburgh, Pittsburgh, PA 15260, USA

**Keywords:** lipidomic profiling, fatty acid oxidation disorders, cardiolipine, sphingomyelins, ceramides

## Abstract

Long-chain fatty acid oxidation disorders (lc-FAOD) are a group of diseases affecting the degradation of long-chain fatty acids. In order to investigate the disease specific alterations of the cellular lipidome, we performed undirected lipidomics in fibroblasts from patients with carnitine palmitoyltransferase II, very long-chain acyl-CoA dehydrogenase, and long-chain 3-hydroxyacyl-CoA dehydrogenase. We demonstrate a deep remodeling of mitochondrial cardiolipins. The aberrant phosphatidylcholine/phosphatidylethanolamine ratio and the increased content of plasmalogens and of lysophospholipids support the theory of an inflammatory phenotype in lc-FAOD. Moreover, we describe increased ratios of sphingomyelin/ceramide and sphingomyelin/hexosylceramide in LCHAD deficiency which may contribute to the neuropathic phenotype of LCHADD/mitochondrial trifunctional protein deficiency.

## 1. Introduction

Long-chain fatty acid oxidation disorders (lc-FAOD) are inherited metabolic diseases affecting the degradation of chain fatty acids with a chain length >C12. During acute episodes of metabolic decompensation, the incomplete oxidation of long chain fatty acids results in the accumulation of toxic acyclcarnitines and energy deficiency due to impaired production of redox equivalents for oxidative phosphorylation [1]. Defects of lc-FAO include enzymes of the carnitine cycle and mitochondrial β-oxidation, namely carnitine palmitoyltransferase deficiencies (CPT I and CPT II), very long-chain acyl-CoA dehydrogenase (VLCAD) deficiency, long-chain 3-hydroxy-acyl-coA dehydrogenase (LCHAD) deficiency, and mitochondrial trifunctional protein (MTP) deficiency. The development of tandem mass spectrometry for the analysis of acylcarnitines in dried blood spots (DBS) enabled a valuable and fast screening for lc-FAOD based on a specific acylcarnitine pattern for each disease [2,3]. These metabolites result from impaired metabolism of lc-fatty acids in organs and tissues due to mitochondrial leakage or active transport of accumulating metabolites by the carnitine shuttle towards the cytosol, and can be easily measured in DBS [4]. This same technology allows newborn screening for these autosomal recessive diseases, and accumulated data from many newborn screening programs worldwide identify a total incidence for fatty acid oxidation disorder of about 1:9.300 newborns [5]. Treatment of lc-FAOD includes avoidance of fasting as well as a fat-restricted and fat-modified diet in which lc-dietary fatty acids are replaced in part by medium-chain fatty acids (MCT oil) [6,7].

Enzymes of mitochondrial β-oxidation are organized in a multi-protein complex that also interacts with the mitochondrial supercomplexes of the respiratory chain [8]. Therefore, the interaction with mitochondrial membranes and their lipids is a requisite for several mitochondrial functions. It has previously been described that the α-subunit of the trifunctional protein is directly involved in the remodeling of mitochondrial cardiolipins [9,10]. In addition, the deacetylation of specific lysine residues allows binding of the VLCAD enzyme to cardiolipin at the inner mitochondrial membrane to ensure proper FAO flux [11]. We have recently shown in a murine model of VLCAD deficiency (VLCAD^−/−^ mouse) an alteration of the whole cellular lipidome affecting cellular functions [12]. Plasma metabolomic analysis in humans also suggested an alteration of complex lipids pathway flux [13,14].

In order to further investigate whether lc-FAOD specific disturbances in the degradation of fatty acids results in a disease specific complex lipid profile that contributes to the wide spectrum of symptoms, we performed comprehensive undirected lipidomic analysis in fibroblasts from patients with different lc-FAOD, namely CPT II, VLCADD and LCHADD. With this approach, we have identified a disease specific disturbed lipid composition similar to previous findings in the mouse model.

## 2. Materials and Methods

### 2.1. Cell Culture

Experiments were performed with fibroblasts from four human healthy controls, retrieved either commercially by Coriell Institute (https://www.coriell.org/ (accessed on June 2019).) or by biopsies from patients of Prof. Dr. Jerry Vockley (unpublished) and Prof. Dr. Daniela Karall [15]. Informed consent for research studies was obtained according to institutional guidelines. Detailed information on the cell line and number of biological replicates is reported in Table 1. Skin dermal cells were grown in DMEM (× 1) medium containing 10% fetal bovine serum (FBS), 4.5 g/L D-Glucose, GlutaMAX, 20 Mm HEPES, 100 units/mL penicillin, and 100 μg/mL streptomycin, at 37 °C in air with 5% CO_2_. A severe form of VLCADD was confirmed in the used cell lines by enzyme testing with a method described previously [16]. All analyzed VLCADD cell lines showed a residual activity in the range 0–10% compared to healthy controls.

### 2.2. Lipid Extraction for Mass Spectrometry Lipidomics

Mass spectrometry-based lipid analysis was performed by Lipotype GmbH (Dresden, Germany) as described [17]. Fibroblasts were kept in culture over 6 weeks until the needed amount of cells for analysis was reached. From this culture, 3*10^6^ cells were pelleted for lipidomic analysis. Lipids were extracted using a two-step chloroform/methanol procedure [18]. Samples were spiked with internal lipid standard mixture containing: cardiolipin 16:1/15:0/15:0/15:0 (CL), ceramide 18:1;2/17:0 (Cer), diacylglycerol 17:0/17:0 (DAG), hexosylceramide 18:1;2/12:0 (HexCer), lyso-phosphatidate 17:0 (LPA), lyso-phosphatidylcholine 12:0 (LPC), lyso-phosphatidylethanolamine 17:1 (LPE), lyso-phosphatidylglycerol 17:1 (LPG), lyso-phosphatidylinositol 17:1 (LPI), lyso-phosphatidylserine 17:1 (LPS), phosphatidate 17:0/17:0 (PA), phosphatidylcholine 17:0/17:0 (PC), phosphatidylethanolamine 17:0/17:0 (PE), phosphatidylglycerol 17:0/17:0 (PG), phosphatidylinositol 16:0/16:0 (PI), phosphatidylserine 17:0/17:0 (PS), cholesterol ester 20:0 (CE), sphingomyelin 18:1;2/12:0;0 (SM), triacylglycerol 17:0/17:0/17:0 (TAG). After extraction, the organic phase was transferred to an infusion plate and dried in a speed vacuum concentrator. 1st step dry extract was re-suspended in 7.5 mM ammonium acetate in chloroform/methanol/propanol (1:2:4, *V:V:V*) and 2nd step dry extract in 33% ethanol solution of methylamine in chloroform/methanol (0.003:5:1; *V:V:V*). All liquid handling steps were performed using Hamilton Robotics STARlet robotic platform with the Anti Droplet Control feature for organic solvents pipetting.

### 2.3. MS Data Acquisition

Samples were analyzed by direct infusion on a QExactive mass spectrometer (Thermo Scientific) equipped with a TriVersa NanoMate ion source (Advion Biosciences). Samples were analyzed in both positive and negative ion modes with a resolution of Rm/z = 200 = 280,000 for MS and Rm/z = 200 = 17,500 for MSMS experiments, in a single acquisition. MSMS was triggered by an inclusion list encompassing corresponding MS mass ranges scanned in 1 Da increments [19]. MS and MSMS data were combined to monitor CE, DAG and TAG ions as ammonium adducts; PC, PC O-, as acetate adducts; and CL, PA, PE, PE O-, PG, PI and PS as deprotonated anions. MS only was used to monitor LPA, LPE, LPE O-, LPI and LPS as deprotonated anions; Cer, HexCer, SM, LPC and LPC O- as acetate adducts.

### 2.4. Data Analysis and Post-Processing

Data were analyzed with in-house developed lipid identification software based on LipidXplorer [20,21]. The identified lipid molecules were quantified by normalization to a lipid class specific internal standard. The amounts in pmols of individual lipid molecules (species of subspecies) of a given lipid class were summed to yield the total amount of the lipid class. The amounts of the lipid classes were normalized to the total lipid amount yielding mol % per total lipids.

The quantities of the lipid species containing the same number of double bonds were summed and these values were normalized to the total amount of the given lipid class. The values were thus given as mol % per total lipids. The quantities of the lipid species containing the same number of carbon atoms in the hydrocarbon moiety were summed, and these values were normalized to the total amount per total lipids. Data post-processing and normalization were performed using by Lipotype GmbH an in-house developed data management system (LipotypeZoom Interactive Data and Visualisation software, Dresden, Germany). Only lipid identifications with a signal-to-noise ratio >5, and a signal intensity 5-fold higher than in corresponding blank samples were considered for further data analysis. Statistical parametric and non-parametric analysis were conducted with LipotypeZoom Interactive Data and Visualisation software and further confirmed by the Mann–Whitney test and Friedman test via GraphPad Prism 6.0 (GraphPad Software, San Diego, CA, USA). Differences were considered significant if *p* < 0.05.

### 2.5. Statistical Analysis

All reported data are presented as means ± standard deviation (SD). *n* denotes the number of biological replicates (*n* = 2–7; Healthy controls *n* = 7; VLCADD *n* = 6; LCHADD *n* = 3; CPT2D *n* = 2). Statistical parametric and non-parametric analysis were conducted with LipotypeZoom Interactive Data and Visualisation software and further confirmed by the Mann–Whitney test and Friedman test via GraphPad Prism 6.0 (GraphPad Software, San Diego, CA, USA). Differences were considered significant if *p* < 0.05.

## 3. Results

### 3.1. Long-Chain Fatty Acid Oxidation Disorders (lc-FAOD) Result in Different Alteration of the Cellular Lipidome

**Figure 1:**
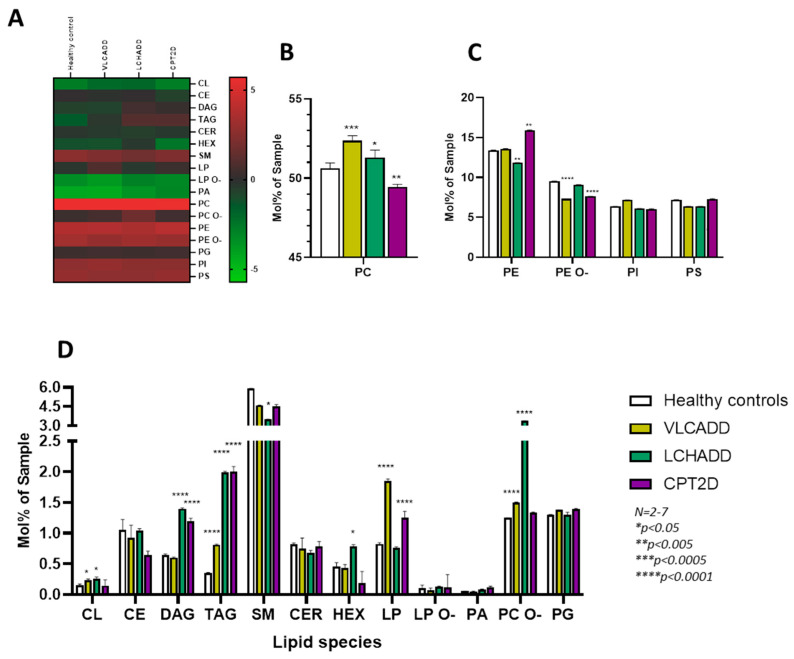
Disease specific alteration of lipid composition. (**A**) Heatmap of log2 transformed lipids concentration from fibroblasts of healthy controls and from VLCADD, LCHADD and CPT2 patients under control conditions (2 fold enrichment, *p*-value < 0.05). (**B**–**D**). Quantification of different lipid classes in fibroblasts from of healthy controls and from VLCADD, LCHADD and CPT2 patients under control conditions. Mol % sample: indicates the moles of the lipid species extracted from one sample calculated as percentage of the total amount of lipid extract. PC: Phosphatidylcholine; PE: Phosphatidylethanolamine; PE O-: Phosphatidylethanolamine (-ether); PI: Phosphatidylinositol; PS: Phosphatidylserine; SM: Sphingomyelins; CL: Cardiolipins; CE: Cholesterol Ester; DAG: Diacylglycerides; TAG: Triacylglycerides; CER: Ceramides; HEX: Hexosylceramides; LP: Lysophospholipids; LP O-: Lysophospholipids (-ether); PA: Phosphatidic acid; PC O-: Phosphatidylcholine (-ether); PG: Phosphatidylglycerol. Values denoted by * were considered significant if *p* < 0.05 (Two way ANOVA; Mann–Whitney test; Friedman test and Tukey’s Test). * indicates significant differences between healthy controls and diseases.

Previous reports have shown that fibroblasts from VLCAD^−/−^ mice have an altered lipidome [12]. To investigate whether this effect also occurs in fibroblasts from patients with lcFAOD we performed a comprehensive undirected lipidomic analysis in cell lines from CPT2, VLCADD and LCHADD patients. In total, more than 1450 lipid species of 23 classes could be identified. A comprehensive summary of log2 transformed values is shown in Figure 1.

We observed disease-specific differences in the phospholipid profile (Figure 1). In particular, the content of phosphatidylcholine (PC) was significantly higher in VLCADD and LCHADD cell lines compared to healthy controls (52.4 mol % sample ± 10.9 and 51.3 mol % sample ± 14.1 vs. 50.6 mol % sample ± 11.6), whereas it was significantly reduced in CPT2D cell lines (Figure 1B). On the other hand, the content of phosphatidylethanolamine (PE) was significantly lower in LCHADD cells (11.8 mol % sample ± 0.34 vs. 13.4 mol % sample ± 0.33), but significantly increased in CPT2D cell lines compared to healthy controls (16 mol % sample ± 0.8 vs. 13.4 mol % sample ± 0.33) (Figure 1B). Alteration in the molar ratio of PC/PE has been described in several diseases [22,23,24] and alteration of phospholipid composition in mitochondrial membranes has been proposed to modulate energy metabolism [25]. As shown in Table 2, the PC/PE ratio was unaffected in cells from VLCADD patients, but increased in LCHADD cell lines and remarkably reduced in CPT2D fibroblasts, suggesting reduced ATP production and impairment of the electron transfer chain [25]. In keeping with previous reports on VLCAD^−/−^ mice [12], we observed a significantly higher content of the plasmalogen PC ethers (PC O-) in VLCADD cell lines and a remarkable 3-fold higher concentration in LCHADD cells compared to healthy controls (3.38 mol % sample ± 0.17 vs. 1.25 mol % sample ± 0.03) (Figure 1C).

With special regard to sphingomyelin (SM), ceramide (CER) and hexosylceramides (HEX) (Figure 1D), only cell lines from LCHADD patients showed significant differences characterized by a reduction of SM (Figure 1A) and a nearly two-fold increase of HEX (0.79 mol % sample ± 0.14 vs. 0.45 mol % sample ± 0.31) (Figure 1D), suggestive of a redirection of the sphingomyelin-ceramide pathway towards HEX. As was seen in VLCAD^−/−^ mice [12,26], fibroblasts from lc-FAOD patients showed a remarkable accumulation of storage lipids. As depicted in Figure 1D, LCHADD and CPT2D cell lines in particular displayed a two-fold higher accumulation of diacylglycerides (DAG) and a six-fold higher content of triacylglycerides (TAG) compared to healthy controls.

Due to the role of HADHA in cardiolipin remodeling, it was not surprising to find a two-fold higher content of total cardiolipins in LCHADD; however, a significantly higher content of this lipid class was also detected in VLACDD cell lines (Figure 1D). No alteration in the cardiolipin content was found in CPT2D cells.

As shown in Figure 1D, there was a significant higher content of bioactive proinflammatory lysophospholipids (LP) in fibroblasts from VLCADD patients compared to healthy controls (1.85 mol % sample ± 0.51 vs. 0.84 mol % sample ± 0.22). A similar increase was also observed in CPT2D cell lines whereas no difference could be detected in fibroblasts from LCHADD patients (Figure 1D).

In keeping with the results reported previously on fibroblast from VLCAD^−/−^ mice [12], we observed sex-related differences in the lipidome of healthy controls and VLCADD cell lines (data not shown) suggesting that sex may indeed play a role in the biosynthesis and remodeling of complex lipids. However, the limited number of samples available made a statistical analysis rather difficult. In contrast to inbred mice strains such as the VLCAD^−/−^ mouse, the remarkable intra-individual genetic variability as well as the different mutations on ACADVL gene carried by the used cell lines may have a deep effect on the lipidomic profile. Therefore, sex-related differences were not taken further into consideration. In summary, defects of mitochondrial β-oxidation do not only affect the degradation of fatty acids, but also result in remarkable alterations of the cellular lipidome. The greatest differences have been observed in LCHADD fibroblasts.

### 3.2. Characterization of Total Fatty Acid Chain Length and Desaturation Degree in lc-FAOD

**Figure 2:**
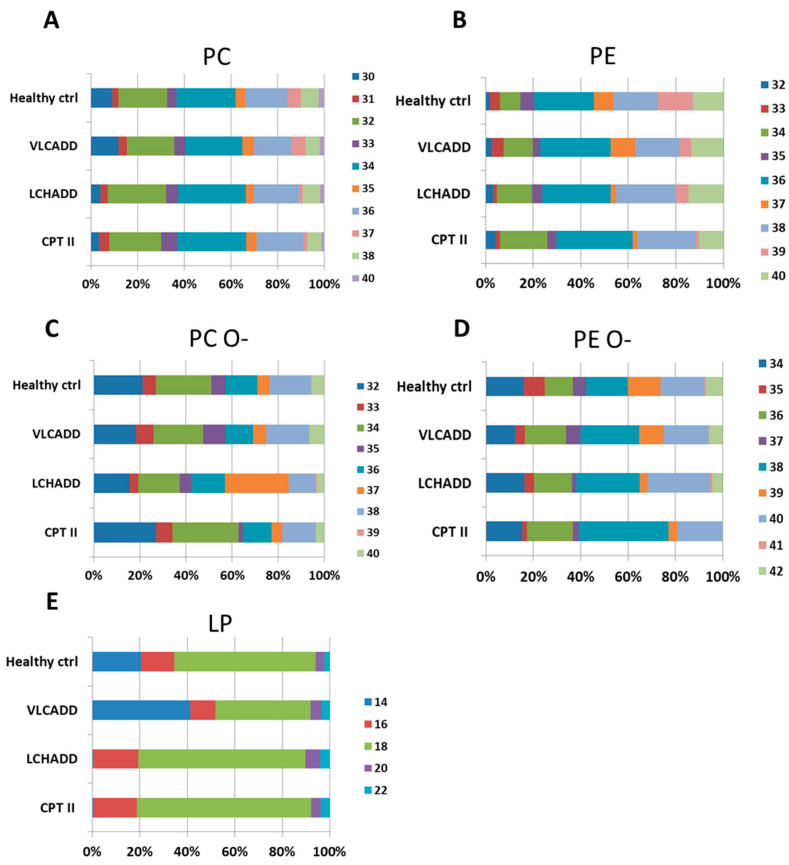
Change of total fatty acid chain length in phosphatidylcholine (**A**), phosphatidylethanolamine (**B**), their corresponding plasmalogens PC O-: Phosphatidylcholine (-ether) (**C**) and PC O-: Phosphatidylethanolamine (-ether)-(**D**) and lysophospholipids (**E**). Concentrations of the measured fatty acid chain length are represented as percentages. Mol % sample: indicates the moles of the lipid species extracted from one sample calculated as percentage of the total amount of lipid extract.

In order to investigate whether lc-FAOD induces changes in different lipid species, we investigated PC and PE as the most represented lipid classes along with the corresponding plasmalogens. An overview of the total fatty acid chain length in fibroblasts from healthy controls and from lc-FAOD patients is summarized in Figure 2. Among the lc-FAOD cells, the lipidomic profile of VLCADD did not differ from healthy controls in contrast to LCHADD and CPT2D. All analyzed cell lines displayed similar profiles in the total chain length of PC without relevant differences (Figure 2A). LCHADD and CPT2D fibroblasts showed a slight reduction of species with chain length of 30 and 37 carbons with an increase of chain lengths of 32 and 34 carbons. With respect to PE, the total chain length profile showed only minor differences in VLCADD cells (Figure 2B), while alterations in LCHADD and CPT2D fibroblasts were more pronounced. Here, we observed a significant reduction of species with a total of 33 and 37 carbons, whereas species with an even number of carbons (34, 36 and 38) were remarkably increased. With regard to the plasmalogen PC O- (Figure 2C), the increase of the total PC O- amount in LCHADD was also reflected in a four-fold increase of species with a total of 37 carbons, likely suggesting the incorporation of the non-metabolized long chain fatty acids into this lipid class. Despite a significant lower amount of PE O- in VLCADD and CPT2D fibroblasts, only the last group displayed differences in the total chain length with a two-fold higher accumulation of C36 and C38 compared to healthy controls. Surprisingly, the most evident disease specific effect was observed in the profile of LP where VLCADD fibroblasts had mostly C14 species, while LCHADD and CPT2D fibroblasts had excess C18 species, paralleled by an accumulation of C14 acylcarnitines in VLCADD and C16/C18 acylcarnitines in LCHADD and CPT2D.

**Figure 3:**
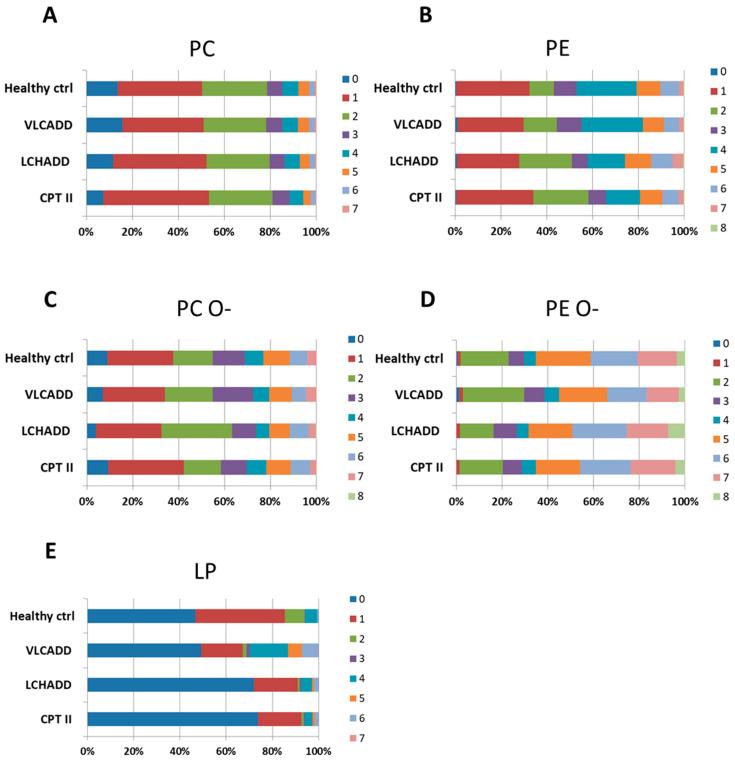
Representation of the desaturation degree in phosphatidylcholine (**A**), phosphatidylethanolamine (**B**), their corresponding plasmalogens PC O-: Phosphatidylcholine (-ether) (**C**) and PC O-: Phosphatidylethanolamine (-ether)-(**D**) and lysophospholipids (**E**). Concentrations of the measured fatty acid chain length are represented as percentages. Mol % sample: indicates the moles of the lipid species extracted from one sample calculated as percentage of the total amount of lipid extract.

In keeping with these results, the desaturation degree PC, PE, PC O- and PC O- did not remarkably change between healthy controls and lc-FAOD (Figure 3A–D). In contrast, the degree of desaturation in LP differed, with a significant increase of the degree of desaturation in VLCADD fibroblasts, whereas saturated LP species were enhanced by about 25% in LCHADD and CPT2D (Figure 3E).

### 3.3. Disease Specific Cardiolipin (CL) Composition in lc-FAOD

**Figure 4:**
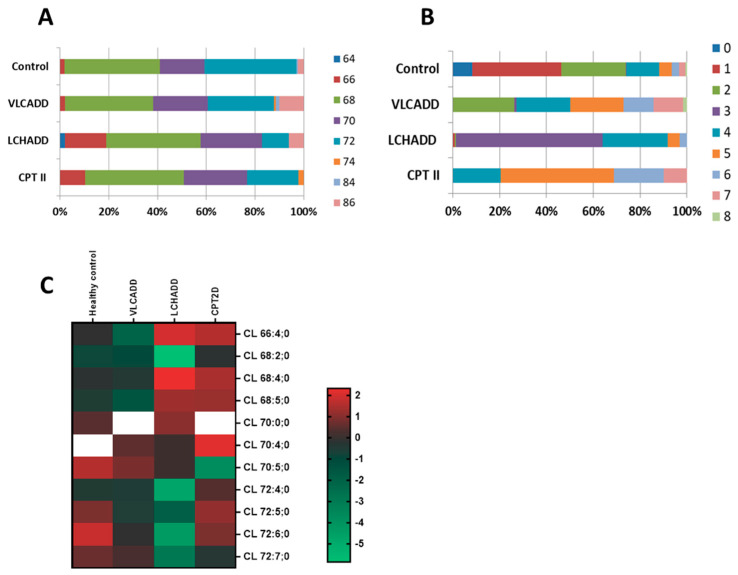
Disease specific alteration of cardiolipins in healthy controls and VLCADD, LCHADD and CPT2D fibroblasts. (**A**) Change of total fatty acid chain length. (**B**) Representation of the desaturation degree. (**C**) Heatmap of log2 transformed concentration of specific cardiolipin species. Concentrations of the measured fatty acid chain length are represented as percentages. Mol % sample: indicates the moles of the lipid species extracted from one sample calculated as percentage of the total amount of lipid extract.

It has recently been recognized that the *HADHA* gene encoding the α-subunit of the human mitochondrial trifunctional enzyme is crucial for cardiolipin remodeling [27]. Dysfunctional cardiolipin composition has been described in diabetic cardiomyopathy, ischemia reperfusion injury and other inherited cardiomyopathies [28]. As enzymes of mitochondrial β-oxidation are physically associated with OXPHOS in a multifunctional mitochondrial protein complex [8,29], we focused specifically on the CL lipid class. The total fatty acid chain length was very similar between healthy controls and VLCADD fibroblasts (Figure 4A). However, LCHADD and CPT2D fibroblasts showed a significant increase of species with a chain length of 66 carbons at the cost of species with longer acyl residues (CL72).

Despite the minor differences in the total fatty acid chain length, the degree of desaturation differed remarkably. About 50% of the CL species of the healthy controls showed a very low degree of desaturation between zero (saturated fatty acids) and two double bonds (Figure 4B). In contrast, fibroblasts from lc-FAOD displayed a much higher degree of desaturation. Saturated species together with CL species with one and two double bonds were fully absent in VLCADD and CPT2D cells, and seen in negligible amounts in the LCHADD cells (Figure 4B). Of particular interest was the fact that in LCHADD, about 90% of the CL species showed a total of 3 or 4 double bonds whereas in CPT2D the degree of desaturation was even higher with 4 to 6 double bonds. These changes were reflected in the CL composition of lc-FAOD fibroblasts (Figure 4C). In healthy controls, the total chain length of acyl residues was higher with a prevalence of CL with >70 carbons. While the CL composition in VLCADD fibroblasts was comparable to healthy controls, there was a remarkable accumulation of shorter CL species with a total carbon number of acyl residues between 66 and 70 in LCHADD. Of note, additional CL species found exclusively in LCHADD fibroblasts were present, namely CL 64:3;0 and CL 64:4;0, strongly suggestive of the higher incorporation of the non-metabolized C16 into CL species (Supplementary Materials). A similar change was found in CPT2D cells with an increase of CL C66 and C68, suggestive of the incorporation of C16 and C18 fatty acids in this lipid class (Figure 4C). Due to the applied method for lipidomic analysis, a detailed evaluation of the acyl residues, as well as the ratio between monolysocardiolipins and mature CL, was not possible.

### 3.4. Sphingolipid Metabolic Flow

**Figure 5:**
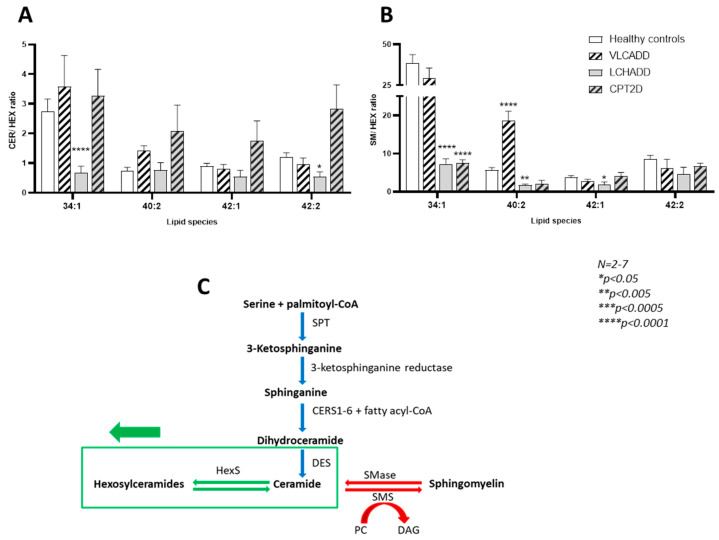
Effect of lc-FAOD on (**A**) ceramide/hexosylceramide and (**B**) sphingomyelin/hexosylceramide ratios for 34:1, 40:2, 42:1 and 42:2 species. (**C**) Schematic representation of sphingolipid metabolism with our working hypothesis on the upregulated biosynthesis of hexosylceramides in LCHADD fibroblasts (Adapted from [31]). Values denoted by * were considered significant if *p* < 0.05 (Two way ANOVA; Mann–Whitney test; Friedman test and Tukey’s Test). * indicates significant differences between healthy controls and diseases.

As alterations in CER and SM composition are associated with several diseases [30], we investigated whether our data was suggestive of an upregulation of the biosynthesis of hexosylceramides. We calculated the ceramide/hexosylceramide (CER/HEX) and sphingomyelin/hexosylceramide (SM/HEX) ratio to evaluate the direction of the sphingolipid metabolic flow as recently described [31]. As represented in Figure 5A, the CER/HEX ratio for the specific species 34:1, 40:2, 42:1 and 42:2 showed a trend towards increase in the ratio of these lipid species in VLCADD and CPT2D fibroblasts. With regard to the SM/HEX ratio, only the species 40:2 in VLCADD fibroblasts was three-fold higher compared to healthy controls. However, in LCHADD fibroblasts, we observed the opposite situation. Specifically, the species 34:1 and 42:2 for the CER/HEX ratio, as well as 34:1, 40:2 and 42:1 for the SM/HEX ratio were significantly reduced strongly suggesting a stimulation of the metabolic flow towards the biosynthesis of hexosylceramides in LCHADD fibroblasts (Figure 5C).

## 4. Discussion

Lc-FAOD are characterized by the accumulation of acylcarnitines of specific chain lengths with toxic effects on tissues and organs [32]. In the present study, we show that monogenic defects of the degradation of long-chain fatty acids (lc-FA) also result in disease-specific alterations of the cellular lipidome.

A recent report on the characterization of cellular lipids in fibroblasts from VLCAD^−/−^ mice described a significant increment of the PC/PE ratio, likely to cope with the enhanced accumulation of intrahepatic lipids. A similar mechanism has been described in the obese *ob/ob* mouse [12,33]. Overall, abnormalities in cellular PC/PE ratios influence energy metabolism and are associated with progression of several diseases affecting different organ systems, such as liver and heart [34,35,36]. Surprisingly, in human VLCADD fibroblasts the PC/PE ratio was not affected possibly because of the different genotype of different cell lines that may differently affect lipidome, unlike in LCHADD fibroblasts carrying the same homozygous mutation.

The increased PC/PE ratio in LCHADD is consistent with deregulated mitochondrial biogenesis [25], as previously described in LCHADD fibroblasts [15], although ATP production can be maintained by the compensatory upregulation of the glycolytic pathway [15]. One of the proposed pathophysiologic mechanisms associated with higher PC/PE ratio is the inhibition of the calcium transport activity of sarco/endoplasmic reticulum Ca2+-ATPase (SERCA) [37,38] contributing to protein misfolding and endoplasmic reticulum stress [33]. This possibility correlates well with the marked increase of PC O- especially in LCHADD fibroblasts. Indeed, calcium signaling is one of the most relevant aspects of the physiological and functional interactions between mitochondria and endoplasmic reticulum [39,40,41]. In contrast to LCHADD fibroblasts, CPT2D cell lines showed a reduced PC/PE ratio. In skeletal muscle, SERCA is responsible for the translocation of calcium ions into the lumen of the sarcoplasmic reticulum and thereby muscle contraction [42,43]. Being the major source of extracellular matrix, fibroblasts account for maintaining a proper muscle structure and function [44]. We may therefore speculate that lipid remodeling leading to increased cellular PE content might serve as compensatory/adaptive mechanism to sustain muscle contraction.

Cardiolipins (CLs) are a class of lipids occurring exclusively in the mitochondrial membrane, where they account for up to 15–20% of the total mitochondrial phospholipids, especially in the inner mitochondrial membrane [45]. There is accumulating evidence for an essential role of this lipid class in several mitochondrial functions. CLs mediate the regulation of the fusion of the mitochondrial inner membrane through the biogenesis and assembly of a dynamin-related GTPase (Opa1) [46]. Aberrant CL composition induces disturbances of mitochondrial dynamics of fusion and fission processes [46,47,48]. In line with this, LCHADD fibroblasts had a higher CL content associated with CL remodeling towards shorter chains likely due to non-metabolized fatty acids which get incorporated into CL species. We speculate that this derangement contributes to the dysregulation of mitochondrial dynamics and reduced respiration described in LCHADD but not VLCAD fibroblasts [15]. Beyond the alteration of the fusion/fission machinery, CLs are also tightly involved in the regulation of mitochondrial metabolism [23,28,45,49,50,51]. Since the α-subunit of MTP is required for CL remodeling, LCHADD/MTPD induced changes in CL composition likely leads to instability of mitochondrial supercomplexes, explaining impairment of previously described mitochondrial functions [15]. An additional difference in lc-FAOD cells was the specific higher content of LP in VLCADD and CPT2D fibroblasts. The presence of these compounds is consistent with enhanced inflammatory response in these diseases [13]. Lipotoxicity due to accumulated long-chain acylcarnitines has been proposed as a mechanism of chronic and subclinical inflammation associated with lc-FAOD [52]. Indeed, studies on VLCAD^−/−^ mice identified an inflammation as defined by an increase in lipid classes involved in inflammatory response signaling cascades [12,26,53,54,55]. It is also important to highlight the altered composition of LP that clearly reflects the incorporation of the accumulated fatty acids. These findings imply that fatty acids with specific chain length not only accumulate as long-chain acylcarnitines in cells, but also participate in remodeling processes that alter the composition and the profile of bioactive lipids [13] and subsequently trigger the onset of symptoms. The composition of SM reported in lc-FAOD fibroblasts supports this hypothesis. SM and CER are important components of cell membranes, but also act as messengers in the inflammatory response [56,57,58]. Due to the structural role of this lipid class in the membrane compartmentalization of transporters and receptors [59,60,61], remodeling of their composition may severely alter cellular functions [62,63]. Our results show that although lc-FAOD did not have a major impact on total chain length of SM acyl residues, the dramatic change in degree of desaturation reflects the impact of these disorders on bioactive lipids [64], and strongly supports a role for inflammation in lc-FAOD as previously suggested [13,52,65]. The largest change occurred in LCHADD fibroblasts in which the composition of SM was shifted towards shorter chain species. In addition, a significant reduction of SM accompanied by a concomitant increase of HEX strongly indicates that the SM metabolic flux promotes increased biosynthesis of the latter lipids in these cells. These findings are extremely relevant as the increase of ceramide formation from SM is known to be specifically detrimental to neurons and oligodendrocytes [66], and elevation of HEX has been implicated in the onset and development of neurodegenerative diseases [67,68,69]. Finally, it is possible that accumulating toxic hydroxyacids in LCHADD/MTPD result in oxidative stress [70,71] that induces mitochondrial damage and the subsequent activation of the sphingomyelin-ceramide pathway involved in the development of peripheral neuropathy in LCHADD/MTPD [66].

## 5. Conclusions

In summary, our data demonstrate that monogenic diseases of lc-FAOD not only affect fatty acid degradation, but also induce a systemic alteration of the composition of complex lipids, resulting in a different lipid profiles between diseases. Importantly, in all investigated diseases, we identified significant remodeling of the acyl residues of mitochondrial cardiolipins affected both chain length and degree of desaturation. Moreover, the aberrant PC/PE ratio, the increased content of plasmalogens as well as of LP strongly support the theory of an inflammatory process in lc-FAOD due to aberrant membrane compartmentalization of transporters and receptors that lead to dysregulation of signaling cascades. We additionally describe here the novel finding of increased ratios of SM/CER and SM/HEX that may contribute to the neuropathic phenotype in LCHADD/MTPD. Overall, this lipidomic approach widens our understanding of the complexity of disease pathogenesis, even though further research is necessary to fully understand the impact of these findings.

## Figures and Tables

**Table 1:** Detailed information on the cell line used in this study.

Disease	Origin	Sex	Allele 1	Allele 2
Healthy control	Coriell Institute (GM04501)	m	WT	WT
Healthy control	Coriell Institute (GM04505)	f	WT	WT
Healthy control	Coriell Institute (GM07492)	m	WT	WT
Healthy control	Coriell Institute (GM08399)	f	WT	WT
Healthy control	Coriell Institute (GM08400)	f	WT	WT
Healthy control	Coriell Institute (GM23964)	m	WT	WT
Healthy control	Coriell Institute (GM23976)	m	WT	WT
VLCAD	Coriell Institute (GM06127)	m	c.925G > a	c.925G > A
VLCAD	Coriell Institute (GM09093)	f	c.515T > C	c.637G > A
VLCAD	Coriell Institute (GM17475)	m	c.364A > G	c.364A > G
VLCAD	Prof. Dr. J. Vockley	f	c.1619T > C	c.1707_ 1715dupAGACGGGGC
VLCAD	Prof. Dr. J. Vockley	m	c.520G > A	c.1825G > A
VLCAD	Prof. Dr. J. Vockley	f	c.14T > C	c.1182 + 2dupT
LCHAD	Prof. Dr. D. Karall	m	c.1528G < C	c.1528G < C
LCHAD	Prof. Dr. D. Karall	f	c.1528G < C	c.1528G < C
LCHAD	Prof. Dr. D. Karall	m	c.1528G < C	c.1528G < C
CPT II	Coriell Institute (GM17413)	f	c.338T > C	c.338T > C
CPT II	Prof. Dr. J. Vockley	m	c.338T > C	c.338T > C

M: Male; f: Female.

**Table 2:** Phosphatidylcholine to phosphatidylethanolamine ratio measured in fibroblasts from healthy controls and from VLCADD, LCHADD and CPT2D patients.

Disease	PC/PE Ratio
Healthy controls	3.78
VLCADD	3.85
LCHADD	4.33
CPT2	3.11

## Data Availability

The data presented in this study are available on request from the corresponding author. The data are not publicly available as they contain original non published data.

## Supplementary Materials

The following are available online at https://www.mdpi.com/article/10.3390/cells10051239/s1, Figure S1: Heatmap of log2 transformed concentration of measured cardiolipin species in healthy controls and lc-FAOD.
