# Supplementary material for: Different Lipid Signature in Fibroblasts of Long-Chain Fatty Acid Oxidation Disorders
Source: Cells. 2021 May 18;10(5):1239. doi: 10.3390/cells10051239 (PMC8157847; doi:10.3390/cells10051239)
Supplement: Supplementary file 1 [file cells-10-01239-s001.zip › cells-1202152-supplementary.pdf]

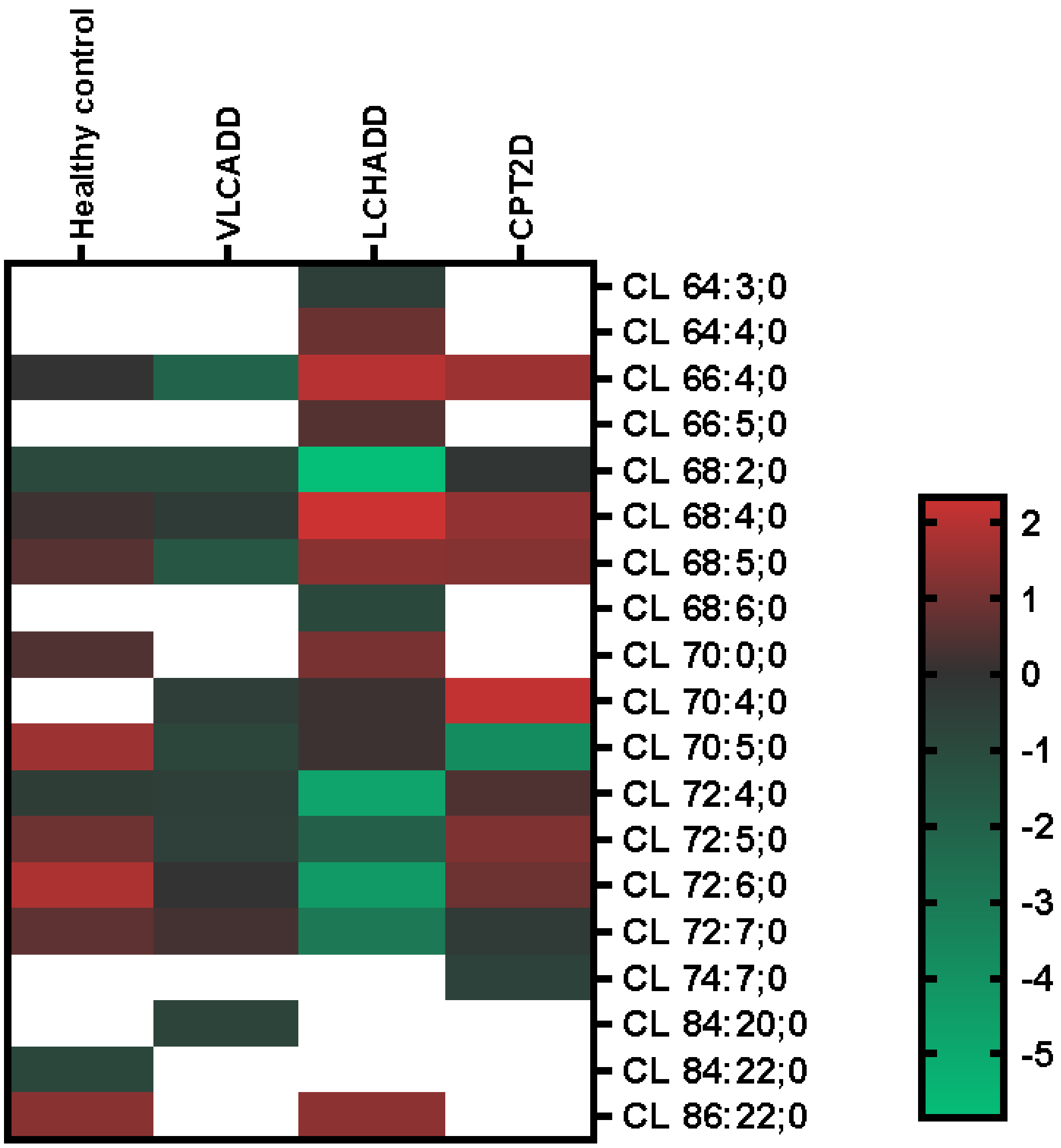

Figure S1. Heatmap of log2 transformed concentration of measured cardiolipin species in healthy controls and lc-FAOD.
